# Leakage-aware race-level upset-risk diagnostics for prospective horse-race prediction

**DOI:** 10.3389/frai.2026.1922250

**Published:** 2026-08-17

**Authors:** Shuichi Sugiura

**Affiliations:** Graduate School of Psychology, Kansai University, Suita, Japan

**Keywords:** calibration, data leakage, horse-race prediction, machine learning, predictive modeling, race-level upset risk, selective-use evaluation, temporal validation

## Abstract

Prospective prediction systems should not only generate individual-level predictions but also indicate when event-level conditions suggest high upset risk or difficult-to-act-on cases. This study developed and evaluated a leakage-aware race-level upset-risk diagnostic for prospective horse-race prediction under temporal validation. Japanese flat-racing data were analyzed using a fixed pre-event evaluation framework. A race-level binary upset label was constructed retrospectively from post-event results, payouts, and final popularity for label construction and evaluation only, whereas prediction features were restricted to pre-event race-structure variables. The temporal split used 2015–2022 races for training, 2023–2024 races for validation, and races from January 5, 2025 to May 10, 2026 for independent testing. Candidate-model assessment was performed using the validation split only, and the primary revised model was a prespecified unweighted standardized logistic regression model. In the temporal test set of 4,556 races, the primary revised model achieved ROC AUC: 0.6459, PR-AUC: 0.6308, Brier score: 0.2325, and log loss: 0.6567. Upset-risk stratification showed that the top 10% highest-upset-probability races had an observed upset rate of 0.6820, compared with a baseline rate of 0.5151. Conversely, the bottom 10% lowest-upset-probability races had an observed upset rate of 0.2193. These findings suggest that a race-level upset-risk diagnostic can stratify races into high-risk and lower-risk strata while remaining separate from horse-level prediction scores. The proposed framework emphasizes prediction-time information constraints, leakage prevention, validation-only candidate-model assessment, calibration assessment, and selective-use evaluation as practical components of deployment-oriented machine-learning evaluation.

## Graphical Abstract

**Figure:**
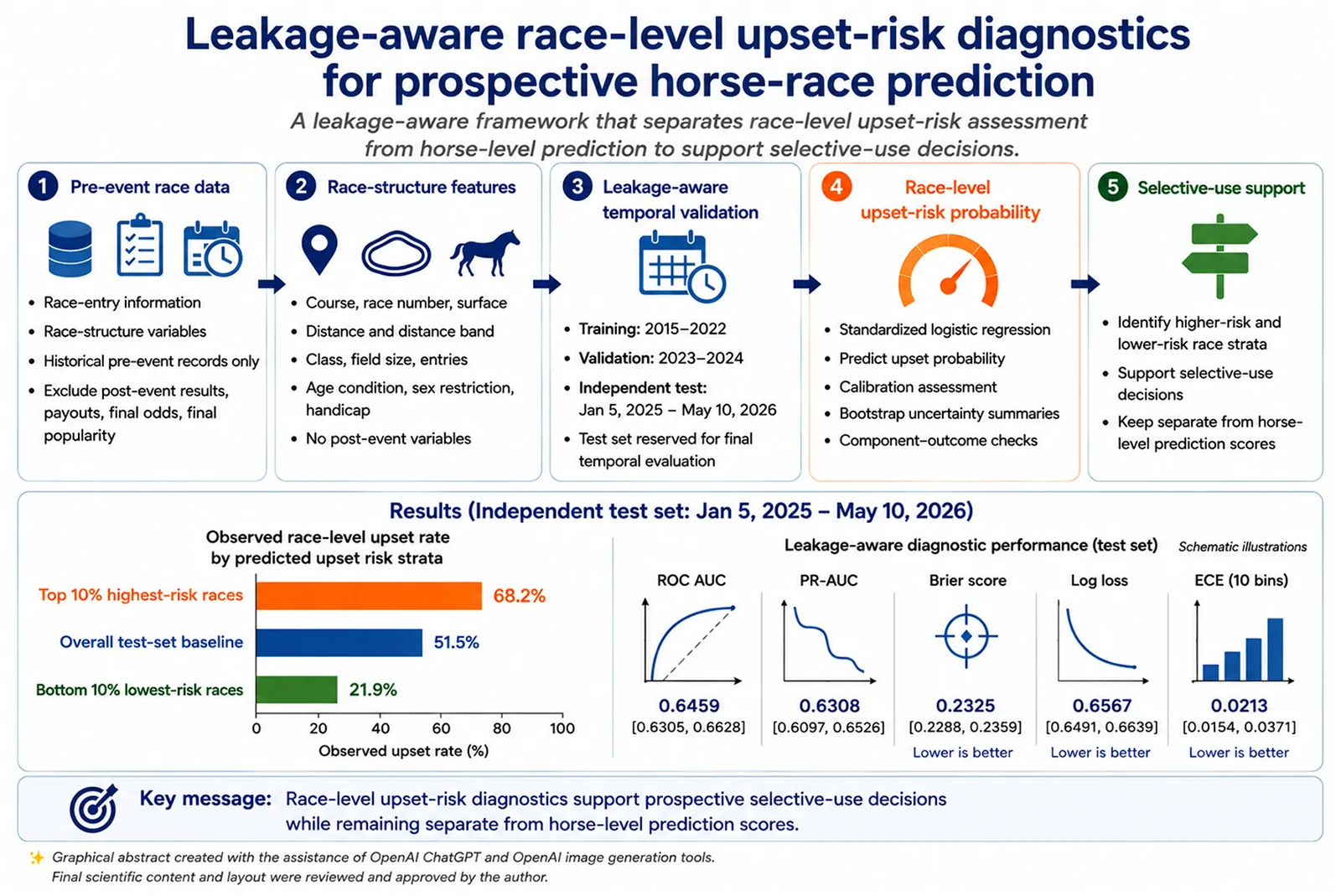

## 1 Introduction

Machine-learning systems for prospective prediction are often evaluated by their ability to assign accurate probabilities or rankings to individual outcomes. However, deployment-oriented prediction also requires identifying cases in which predictions may be uncertain, unstable, or difficult to act upon. Selective prediction and classification with a reject option formalize this problem by allowing a model to abstain or to restrict predictions to cases for which its predictions are sufficiently reliable (Chow, 1970; El-Yaniv and Wiener, 2010; Geifman and El-Yaniv, 2017; Hendrickx et al., 2024). This perspective is relevant when model users need not only a point prediction but also a diagnostic indication of when prediction itself is difficult.

Horse-race prediction provides a useful setting for studying this issue. Earlier horse-race studies examined statistical handicapping and winner prediction, including multinomial logit modeling and support-vector-machine classification for competitive-event outcomes (Bolton and Chapman, 1986; Lessmann et al., 2009). Broader sports-prediction reviews have summarized feature engineering, classifier selection, and evaluation procedures for sport result prediction (Bunker and Thabtah, 2019; Horvat and Job, 2020). Recent sports-AI literature further shows that artificial intelligence is increasingly used across sports analytics, including sensing, tracking, time-series analysis, and match or event outcome prediction (Li et al., 2025), and recent deep-learning work illustrates the continued focus on improving sport event outcome prediction using structured match data and neural architectures (Gao et al., 2025). In contrast, the present study does not aim to optimize horse-level rankings or betting returns; instead, it evaluates whether pre-event race-structure variables can stratify races by race-level upset risk under leakage-aware temporal validation.

Evaluation of such systems must also guard against data leakage. If post-event information such as final results, payouts, final odds, or final popularity enters the feature construction process, model performance can be overestimated (Kaufman et al., 2012). Temporal validation is also important because random splits may be optimistic when data have temporal or hierarchical dependence (Roberts et al., 2017). Race-level upset risk is not equivalent to epistemic uncertainty of the prediction model; in this study, it is treated as an operational proxy for event-level instability and as a target for upset-risk stratification. Within this leakage-aware evaluation perspective, the present study addresses a race-level question: whether a separate diagnostic can identify higher-risk and lower-risk race strata without changing horse-level prediction scores.

The objective of this study was to construct and evaluate a race-level upset-risk diagnostic for prospective horse-race prediction. The diagnostic was evaluated under temporal validation using a fixed data cutoff date, validation-only candidate-model assessment, calibration assessment, and selective-use analysis. The diagnostic output was not integrated into horse-level scores; instead, it was evaluated as a separate decision-support layer for upset-risk stratification and selective-use support.

## 2 Materials and methods

### 2.1 Data source, prediction setting, and temporal validation

The analysis used Japanese flat-racing data processed for a prospective prediction pipeline. The source data were derived from JRA-VAN Data Lab. records (JRA-VAN, 2025). The intended prediction setting was after race-entry information was available and before race outcomes, payouts, final odds, and final popularity were known. The data cutoff was fixed at May 10, 2026 to align the race-level diagnostic with the available pre-event analysis base. The input sources were processed race results, processed payout records, and race-entry feature bases derived from race-entry information and historical pre-event records. The temporal split was defined as 2015–2022 for training, 2023–2024 for validation, and January 5, 2025–May 10, 2026 for independent temporal testing.

The race-level dataset contained 37,837 merged races. The training, validation, and test splits contained 26,625, 6,656, and 4,556 races, respectively. Random splitting was not used. Model selection was performed using the validation split only, and the temporal test split was used only for final evaluation. The temporal split manifest is provided in Supplementary Table S3.

**Figure 1:**
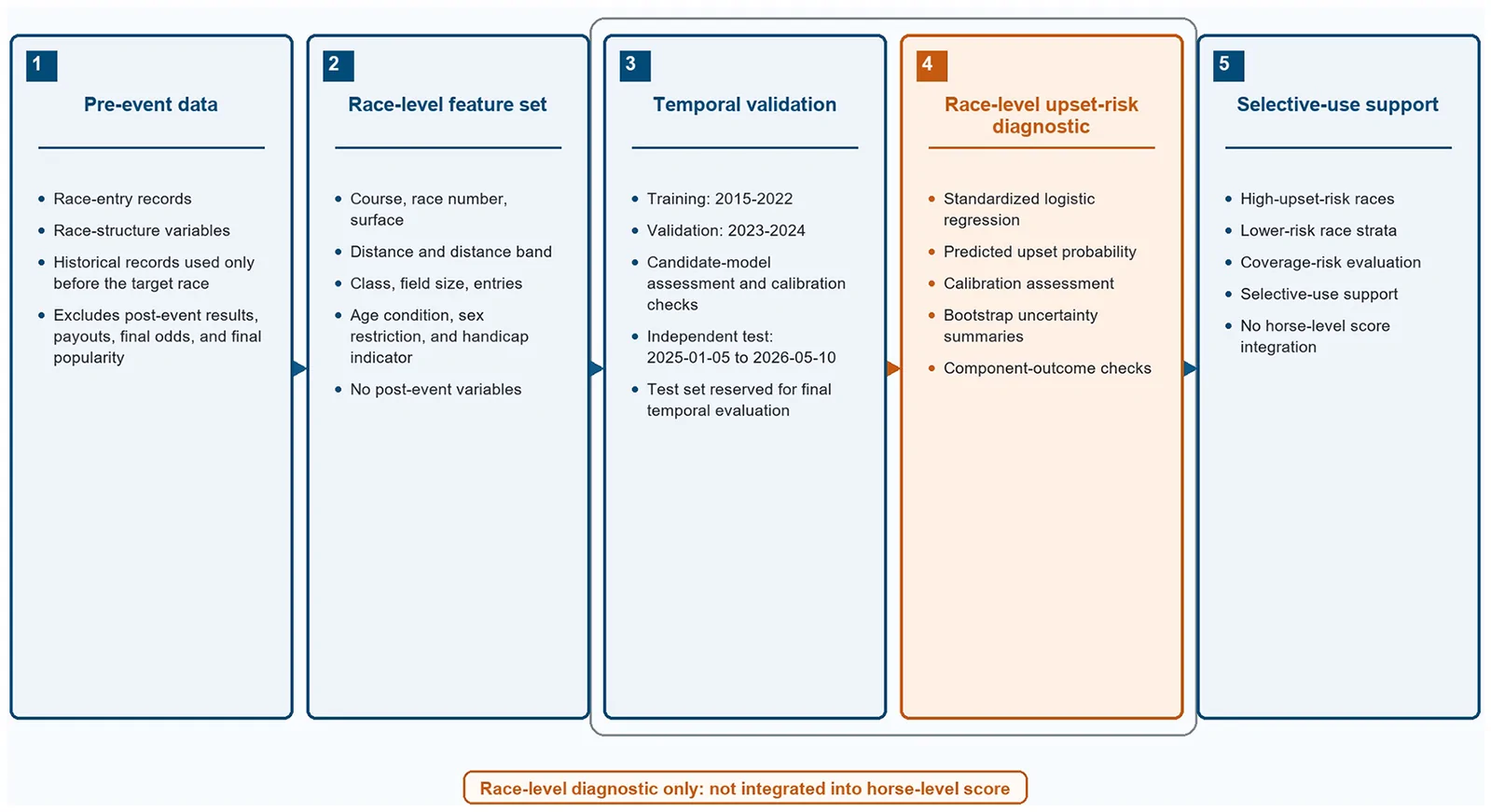
Workflow for evaluating a race-level upset-risk diagnostic alongside horse-level prediction models under temporal validation. Horse-level prediction models were kept separate from the race-level diagnostic. The revised race-level model used prediction-time available race-structure variables, including field size, course, surface, distance band, and class. A separate diagnostic model estimated race-level upset risk and was evaluated using discrimination, calibration, bootstrap uncertainty summaries, and upset-risk stratification. The race-level diagnostic was not integrated into the horse-level prediction score; instead, it was used to support selective-use decisions by identifying higher-risk and lower-risk race strata.

The overall workflow is shown in Figure 1. Pre-event race-structure variables were used to construct race-level diagnostic features. The resulting race-level diagnostic was evaluated as a separate upset-risk stratification layer and was not integrated into the horse-level prediction score.

### 2.2 Race-level outcome label

The primary race-level binary outcome label was target_race_upset. This label was constructed retrospectively from post-event results, payouts, and popularity information for label construction and evaluation only. It was positive if any of three component labels was positive: target_upset_win, target_upset_place, or target_high_payout. The label was intended as an operationally interpretable diagnostic endpoint rather than a universal definition of an upset race.

target_upset_win was positive when the winning horse had final popularity rank 6 or worse, or when the win return was at least 1,000 yen. target_upset_place was positive when a horse with popularity rank 8 or worse finished in the payout-eligible place range under JRA place-betting rules (Japan Racing Association, 2026), when the maximum place return was at least 500 yen, or when a longshot finished in the money. target_high_payout was positive when the race-level exotic payout was at or above the 80th-percentile diagnostic threshold estimated from the training split only. The training-split 80th-percentile threshold was 120,162 yen and was then applied unchanged to validation and test races. This threshold was used only for retrospective outcome-label construction and evaluation and was not used as a prediction feature. target_race_upset was defined as the union of these labels.

Final results, payouts, final odds, and final popularity were not used as prediction features. They were used only to define historical race-level outcome labels and to evaluate model performance.

Detailed outcome-label definitions and the high-payout threshold comparison are provided in Supplementary Table S1.

### 2.3 Race-level feature construction

Race-level features were constructed from pre-event race-structure variables. The primary feature set included race number, racecourse, surface, distance, distance band, class, field size, number of entries, age condition, sex restriction, and handicap indicator when available. These variables were treated as prediction-time available race-entry information.

The revised analysis did not use post-event results, payouts, final odds, final popularity, or same-day uncertain going or weather as prediction features. It also did not rely on horse-level prediction probabilities as primary predictors for the race-level diagnostic. This revision was adopted to keep the diagnostic interpretable and to avoid ambiguity about whether horse-level probabilities had been generated entirely out of sample. The race-level diagnostic features were not fed back into horse-level scores, horse-level ranks, recommendation counts, or calibrated horse-level probabilities.

The feature inventory and leakage audit are provided in Supplementary Tables S2a, b.

### 2.4 Model development and validation-only model selection

Three race-level binary classifiers were compared: a HistGradientBoostingClassifier, standardized logistic regression, and class-balanced standardized logistic regression. The models were implemented using scikit-learn (Pedregosa et al., 2011). Candidate models were fitted using the training split. Because the composite upset outcome was nearly balanced and class weighting affected calibration, the primary revised model was a prespecified unweighted standardized logistic regression model using the full race-structure feature set without calendar year. For transparency, the validation-only selection score used in the candidate-model audit was defined as


$$S_{\text{valid}}=0.70\times \text{PR}\text{-AU}{\text{C}}_{\text{valid}}+0.20\times \text{ROC AU}{\text{C}}_{\text{valid}}-0.10\times \text{Brie}{\text{r}}_{\text{valid}}.$$

Higher values indicate better validation performance. This criterion was used as an audit summary for candidate-model comparison before final test-set interpretation. Test metrics and by-year calibration summaries were recorded for audit only and were not used for model selection.

The primary revised model produced a race-level upset probability for each race. The probability was interpreted as a diagnostic output, not as a replacement for horse-level prediction probabilities.

Candidate-model configurations and validation-only assessment results are provided in Supplementary Table S4.

### 2.5 Calibration, upset-risk stratification, and uncertainty summaries

Performance was evaluated using the area under the receiver operating characteristic curve (ROC AUC), the area under the precision-recall curve (PR-AUC), Brier score, and log loss. ROC AUC and PR-AUC were used to assess discrimination, whereas Brier score and log loss were used to evaluate probabilistic prediction. The Brier score is a classical probability-forecast verification metric (Brier, 1950), and proper scoring rules are important for evaluating probabilistic predictions because they penalize poorly calibrated or overconfident probability estimates (Gneiting and Raftery, 2007). Because the revised model outputs a probability of race-level upset risk, calibration was evaluated as a distinct property of probabilistic prediction. Calibration was assessed using 10-bin calibration tables, expected calibration error (ECE), reliability diagrams, calibration slope, calibration intercept, and bootstrap confidence intervals. ECE was computed as the sample-size-weighted mean absolute difference between the mean predicted probability and the observed event rate across 10 equal-width probability bins. This follows calibration literature emphasizing that classifier calibration should be assessed using complementary graphical and numerical summaries rather than a single scalar metric alone (Guo et al., 2017; Dimitriadis et al., 2021; Silva Filho et al., 2023).

Selective-use support was evaluated by ranking races according to the predicted race-level upset probability. High-upset selection used the top 5%, 10%, 15%, 20%, 30%, 40%, and 50% of races. Low-upset selection used the bottom 5%, 10%, 15%, 20%, 30%, 40%, and 50% of races. For each coverage level, the event rate, baseline event rate, lift, and 95% confidence interval were calculated. Event-rate uncertainty was estimated using race-level bootstrap resampling with race_id as the resampling unit. The diagnostic was evaluated as a race-level upset-risk layer and was not integrated into the horse-level score.

### 2.6 Reproducibility and leakage audit

All analyses were conducted in Python 3.13.7 (Python Software Foundation, Wilmington, Delaware, USA). The main software libraries used for data processing and modeling were pandas 3.0.2 pandas Development Team (open-source software), NumPy 2.4.4 NumPy Developers (open-source software), and scikit-learn 1.8.0 scikit-learn Developers (open-source software). The primary random seed was fixed at 2026 for reproducible model fitting and uncertainty summaries.

To address reproducibility, the analysis generated a fixed feature inventory, outcome-label definition table, temporal-split manifest, model-configuration and model-selection table, leakage audit, reproduction commands, and SHA-256 source manifest. These records documented artifact names, generalized artifact categories, SHA-256 hashes, split definitions, label definitions, candidate model families, selected model, evaluation metrics, and the commands used to rebuild the analysis tables, without exposing workstation-specific directory structures. The feature inventory distinguished prediction features from diagnostic labels and excluded post-event variables.

The leakage audit explicitly recorded that outcome labels used post-event information only for label construction and evaluation, while prediction features were restricted to pre-event race-structure variables. Finish position, payout information, final odds, final popularity, same-race outcomes, same-day uncertain going or weather, and same-day horse weight were not used as prediction features. The validation split was used for candidate-model assessment, and the test split was reserved for final temporal evaluation only.

Software versions, reproduction commands, and the revision source manifest are provided in Supplementary Tables S9, S10.

## 3 Results

### 3.1 Race-level model performance

The primary revised model was an unweighted standardized logistic regression model using prediction-time available race-structure variables. In the independent temporal test set of 4,556 races, the positive rate of target_race_upset was 0.5151. The primary revised model achieved ROC AUC 0.6459 [95% CI: 0.6305–0.6628], PR-AUC 0.6308 [0.6097–0.6526], Brier score 0.2325 [0.2288–0.2359], and log loss 0.6567 [0.6491–0.6639] (Table 1). Validation and test performance were similar in magnitude, suggesting that the model retained modest race-level discrimination under temporal testing.

**Table 1:** Temporal test-set performance of the race-level diagnostic model.

Metric	Estimate	95% confidence interval
Number of races	4,556	—
Positive rate	0.5151	—
ROC AUC	0.6459	0.6305–0.6628
PR-AUC	0.6308	0.6097–0.6526
Brier score	0.2325	0.2288–0.2359
Log loss	0.6567	0.6491–0.6639
ECE-10	0.0213	0.0154–0.0371
Calibration intercept	–0.0730	–0.1383 to –0.0122
Calibration slope	1.0663	0.9525–1.1938

Values in brackets indicate 95% bootstrap confidence intervals based on race-level resampling.

### 3.2 Calibration

The 10-bin ECE in the test split was 0.0213. In the 0.4–0.5 predicted-probability bin, the mean predicted probability was 0.4523 and the observed event rate was 0.4601. In the 0.5–0.6 bin, the mean predicted probability was 0.5572 and the observed event rate was 0.5248. In the 0.6–0.7 bin, the mean predicted probability was 0.6388 and the observed event rate was 0.6453. Calibration error was larger in lower-probability bins, but the central probability ranges that contained most races showed moderate agreement between predicted and observed rates. These calibration findings support interpreting the output as a diagnostic probability rather than a deterministic race classification.

The full calibration-bin table is provided in Supplementary Table S5.

### 3.3 Upset-risk stratification and selective-use support

Upset-risk stratification showed that races with high predicted upset probability had a higher observed upset rate than the test-set baseline (Table 2). The top 5% highest-upset-probability races had an event rate of 0.6842, compared with the baseline rate of 0.5151, corresponding to a lift of 1.33. The top 10% highest-upset-probability races had an event rate of 0.6820 and lift of 1.32. The race-level bootstrap 95% confidence interval for the top 10% event rate was 0.6403–0.7259.

**Table 2:** Upset-risk stratification results in the temporal test set.

Selection	Coverage	*N*	Event rate	Baseline	Lift	95% bootstrap CI
High-upset	Top 5%	228	0.6842	0.5151	1.33	0.6184–0.7412
High-upset	Top 10%	456	0.6820	0.5151	1.32	0.6403–0.7259
High-upset	Top 20%	911	0.6674	0.5151	1.30	0.6355–0.6971
Low-upset	Bottom 5%	228	0.1579	0.5151	0.31	0.1096–0.2105
Low-upset	Bottom 10%	456	0.2193	0.5151	0.43	0.1754–0.2544
Low-upset	Bottom 20%	911	0.2942	0.5151	0.57	0.2634–0.3227

Conversely, races with low predicted upset probability had substantially lower observed upset rates. The bottom 5% lowest-upset-probability races had an event rate of 0.1579 and lift of 0.31 relative to baseline. The bottom 10% lowest-upset-probability races had an event rate of 0.2193 and lift of 0.43, with a race-level bootstrap 95% confidence interval of 0.1754–0.2544.

**Figure 2:**
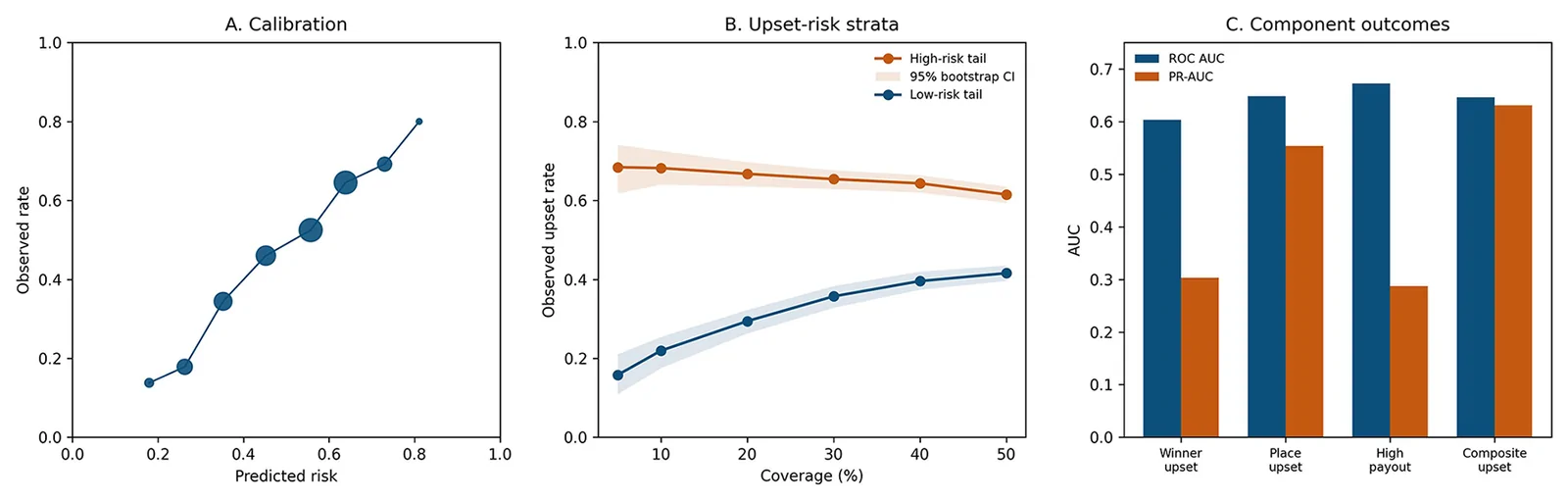
Calibration and upset-risk stratification results for the race-level diagnostic model. **(A)** shows observed upset rates against predicted upset risk in calibration bins. **(B)** shows observed upset rates in high-risk and low-risk subsets across coverage levels; shaded bands indicate 95% bootstrap confidence intervals. **(C)** reports ROC AUC and PR-AUC when the same diagnostic score was evaluated against winner-upset, place-upset, high-payout, and composite-upset outcomes. No common PR-AUC baseline is shown because PR-AUC baselines depend on outcome prevalence.

Figure 2 summarizes calibration, upset-risk stratification, and component-level outcome performance for the revised race-level diagnostic. Full coverage-level stratification results are provided in Supplementary Table S6, and detailed component-level outcome metrics are provided in Supplementary Table S7.

### 3.4 Segment-level patterns

Exploratory segment summaries suggested that upset rates varied by race class and other race-structure variables. In the temporal test set, some higher-class segments had higher observed upset rates than the overall test baseline, whereas maiden races had a lower event rate than the baseline. These segment-level findings were descriptive and were not interpreted causally. They suggest candidate strata for future subgroup-specific calibration or hierarchical upset-risk modeling.

Benchmark and ablation results for race-structure feature sets are provided in Supplementary Table S8.

## 4 Discussion

This study evaluated a race-level upset-risk diagnostic as a separate layer alongside the existing horse-level prediction pipeline. The main finding was that a race-level model using prediction-time available race-structure variables could stratify races into higher-upset-risk and lower-upset-risk strata under temporal validation. Importantly, the diagnostic did not modify horse-level scores or rankings. This separation is methodologically important because it allows upset-risk stratification and selective-use support to be evaluated before any score integration is attempted. The revised analysis should be interpreted as upset-risk stratification, not as estimation of epistemic uncertainty in the horse-level prediction model.

Recent sports-AI literature has emphasized increasingly sophisticated sensing, representation learning, time-series modeling, and outcome-prediction methods (Li et al., 2025; Gao et al., 2025). The revised analysis contributes a complementary perspective: rather than optimizing event-outcome prediction accuracy or horse-level rankings, it evaluates whether pre-event race-structure variables can provide calibrated race-level upset-risk stratification.

The results support the practical relevance of selective-use support in prospective forecasting. The high-upset top 10% of races showed a substantially higher event rate than the temporal test-set baseline, while the low-upset bottom 10% showed a substantially lower event rate. This pattern suggests that race-level upset-risk diagnostics may help distinguish higher-upset-risk race conditions from lower-upset-risk race strata before race outcomes are observed. Consistent with the selective-prediction literature, the diagnostic can be interpreted as a selective-use or coverage-risk tool rather than a direct betting or profit-maximization system.

The study also illustrates the importance of reproducibility and leakage-aware evaluation. The outcome label was constructed from post-event information, but this information was restricted to label construction and evaluation. Prediction features were limited to pre-event race-structure variables. Candidate-model assessment was validation-only, and the test period was reserved for final evaluation. These design choices address common concerns in prediction-model research, including transparent predictor definitions, model configuration, validation procedures, and reproducibility (Collins et al., 2024; Belbasis and Panagiotou, 2022). Deployment-oriented machine-learning systems also require external validation and ongoing monitoring (Yuan, 2024); race-level diagnostics may provide one way to monitor when a forecasting system is operating in a higher-upset-risk regime.

Several limitations should be noted. First, the outcome label target_race_upset is a constructed diagnostic label rather than a universally accepted definition of an upset race. Alternative definitions based on specific payout types, market expectations, or popularity-based outcomes may yield different results. Second, although temporal validation reduces optimistic bias compared with random splitting, the analysis was based on a single racing system and a fixed data cutoff. Third, the diagnostic was evaluated separately from horse-level score integration. Therefore, the present results do not show that incorporating the diagnostic into horse-level scores would improve horse-level predictive accuracy. Fourth, the analysis used historical race data from a licensed data source, and external replication would require access to comparable pre-event and post-event data under the relevant licensing conditions.

Future work should evaluate alternative race-level outcome definitions, subgroup-specific calibration by course or class, and Bayesian models that explicitly represent subgroup-level variation in upset risk. The most conservative next step is to keep the race-level diagnostic separate from the horse-level score and evaluate its use as a selective-use or monitoring layer. Only after such pre-integration evaluation should score integration be considered.

## 5 Conclusion

This study developed and evaluated a leakage-aware race-level upset-risk diagnostic for prospective horse-race prediction under temporal validation. Using prediction-time available race-structure variables, the diagnostic identified higher-upset-risk and lower-upset-risk race strata in an independent temporal test set. The diagnostic was not integrated into horse-level scores, rankings, or recommendation counts. These findings suggest that race-level upset-risk diagnostics may complement horse-level prediction models by supporting upset-risk communication and selective-use decisions under leakage-aware temporal validation.

## Data Availability

The data analyzed in this study is subject to the following licenses/restrictions: the datasets were derived from horse-racing records obtained through JRA-VAN Data Lab. and exported using TARGET frontier JV. Access to these data is governed by licensing agreements with the data providers. Consequently, neither the raw data nor the processed datasets can be publicly released, redistributed, or included in the manuscript or Supplementary materials. However, feature definitions, model specifications, evaluation procedures, and aggregate analysis outputs can be shared without including licensed data. Requests to access these datasets should be directed to JRA-VAN Data Lab (https://jra-van.jp); and TARGET frontier JV (https://jra-van.jp/target/).
