# Supplementary material for: Leakage-aware race-level upset-risk diagnostics for prospective horse-race prediction
Source: Front Artif Intell. 2026 Aug 17;9:1922250. doi: 10.3389/frai.2026.1922250 (PMC13543210; doi:10.3389/frai.2026.1922250)
Supplement: Supplementary file 1 [file Data_Sheet_1.pdf]

Supplementary Material: Leakage-aware race-level upset-risk diagnostics for prospective horse-race prediction

Shuichi Sugiura

This document is the official Supplementary PDF for the manuscript. It provides the race-level outcome-label definitions, feature inventory and leakage audit, temporal split manifest, candidate model configuration and candidate-model assessment, calibration summaries, upset-risk stratification results, software-version and reproduction information, and source manifests. Machine-readable CSV exports are retained in the research package as source files used to construct these appendix tables, but the reviewer-facing supplementary material is this Supplementary PDF.

Licensed raw data and row-level processed datasets are not included in this Supplementary PDF. To avoid exposing local workstation structure, source manifests report artifact names, artifact categories, file sizes, and SHA-256 hashes, but not workstation-specific details.

Supplementary Table S1. Race-level outcome label definitions

Post-event information was used only for outcome-label construction and evaluation, not as prediction features. The high-payout label used a diagnostic threshold estimated from the training split only. The originally submitted all-split 80th-percentile diagnostic threshold was 115,582 yen, whereas the revised training-only 80th-percentile threshold was 120,162 yen. Applying the revised threshold changed 28 temporal-test high-payout labels and only one temporal-test composite `target_race_upset` label.

Label	Level	Type	Definition	Post-event information	Model feature	Allowed use
target__upset__win	race	binary outcome label	winner popularity >= 6 or win return >= 1000 yen	True	False	label construction and evaluation only
target__upset__place	race	binary outcome label	popularity >= 8 in money or max place return >= 500 yen	True	False	label construction and evaluation only
target__high__payout	race	binary outcome label	race exotic payout >= training-split 80th-percentile diagnostic threshold, 120,162 yen	True	False	label construction and evaluation only
target__race__upset	race	binary outcome label	any upset target	True	False	label construction and evaluation only
upset__score__label	race	continuous diagnostic label	mean z-score of log win return, log place return, log exotic payout, favorite miss, and longshot in money	True	False	diagnostic label construction and evaluation only

Supplementary Table S2a. Feature inventory

The feature inventory distinguishes prediction-time race structure used in the revised primary model from diagnostic labels, excluded post-event variables, and candidate horse-level aggregation variables that were not used in the revised primary model. Candidate horse-level aggregation variables were present in the archived feature table but were unavailable or non-informative in the submitted analysis package; therefore, they were excluded from the revised primary model.

Feature	Data type	Role	Prediction-time availability / OOS status	Used in model	Post-event	Note
year	numeric	temporal audit variable	Audit only	False	False	Recorded for temporal audit only and excluded from the revised primary feature set.
course	categorical	pre-event race structure	Available	True	False	Pre-event race-structure information available after race-entry finalization; does not include results, payouts, or final popularity.
race_no	numeric	pre-event race structure	Available	True	False	Pre-event race-structure information available after race-entry finalization; does not include results, payouts, or final popularity.
surface	categorical	pre-event race structure	Available	True	False	Pre-event race-structure information available after race-entry finalization; does not include results, payouts, or final popularity.
distance	numeric	pre-event race structure	Available	True	False	Pre-event race-structure information available after race-entry finalization; does not include results, payouts, or final popularity.
distance_band	categorical	pre-event race structure	Available	True	False	Pre-event race-structure information available after race-entry finalization; does not include results, payouts, or final popularity.
class	categorical	pre-event race structure	Available	True	False	Pre-event race-structure information available after race-entry finalization; does not include results, payouts, or final popularity.
age_condition	numeric	pre-event race structure	Available	True	False	Pre-event race-structure information available after race-entry finalization; does not include results, payouts, or final popularity.
sex_restriction	categorical	pre-event race structure	Available	True	False	Pre-event race-structure information available after race-entry finalization; does not include results, payouts, or final popularity.
handicap	numeric	pre-event race structure	Available	True	False	Pre-event race-structure information available after race-entry finalization; does not include results, payouts, or final popularity.
field_size	numeric	pre-event race structure	Available	True	False	Pre-event race-structure information available after race-entry finalization; does not include results, payouts, or final popularity.
n_rows	numeric	pre-event race structure	Available	True	False	Pre-event race-structure information available after race-entry finalization; does not include results, payouts, or final popularity.
top1_p_y_win	numeric	candidate horse-level aggregation variable	Not verified / excluded	False	False	Candidate diagnostic variable excluded from the revised primary model because it was unavailable or non-informative in the submitted analysis package.
top2_p_y_win	numeric	candidate horse-level aggregation variable	Not verified / excluded	False	False	Candidate diagnostic variable excluded from the revised primary model because it was unavailable or non-informative in the submitted analysis package.
top1_top2_gap	numeric	candidate horse-level aggregation variable	Not verified / excluded	False	False	Candidate diagnostic variable excluded from the revised primary model because it was unavailable or non-informative in the submitted analysis package.
top3_p_y_win_sum	numeric	candidate horse-level aggregation variable	Not verified / excluded	False	False	Candidate diagnostic variable excluded from the revised primary model because it was unavailable or non-informative in the submitted analysis package.
top1_p_y_place	numeric	candidate horse-level aggregation variable	Not verified / excluded	False	False	Candidate diagnostic variable excluded from the revised primary model because it was unavailable or non-informative in the submitted analysis package.
top2_p_y_place	numeric	candidate horse-level aggregation variable	Not verified / excluded	False	False	Candidate diagnostic variable excluded from the revised primary model because it was unavailable or non-informative in the submitted analysis package.

Feature	Data type	Role	Prediction-time availability / OOS status	Used in model	Post-event	Note
p_y_win_entropy	numeric	candidate horse-level aggregation variable	Not verified / excluded	False	False	Candidate diagnostic variable excluded from the revised primary model because it was unavailable or non-informative in the submitted analysis package.
p_y_place_entropy	numeric	candidate horse-level aggregation variable	Not verified / excluded	False	False	Candidate diagnostic variable excluded from the revised primary model because it was unavailable or non-informative in the submitted analysis package.
score_concentration_index	numeric	candidate horse-level aggregation variable	Not verified / excluded	False	False	Candidate diagnostic variable excluded from the revised primary model because it was unavailable or non-informative in the submitted analysis package.
cal_total_score_max	numeric	candidate horse-level aggregation variable	Not verified / excluded	False	False	Candidate diagnostic variable excluded from the revised primary model because it was unavailable or non-informative in the submitted analysis package.
cal_total_score_mean	numeric	candidate horse-level aggregation variable	Not verified / excluded	False	False	Candidate diagnostic variable excluded from the revised primary model because it was unavailable or non-informative in the submitted analysis package.
cal_total_score_sd	numeric	candidate horse-level aggregation variable	Not verified / excluded	False	False	Candidate diagnostic variable excluded from the revised primary model because it was unavailable or non-informative in the submitted analysis package.
cal_rank_score_max	numeric	candidate horse-level aggregation variable	Not verified / excluded	False	False	Candidate diagnostic variable excluded from the revised primary model because it was unavailable or non-informative in the submitted analysis package.
cal_rank_score_gap	numeric	candidate horse-level aggregation variable	Not verified / excluded	False	False	Candidate diagnostic variable excluded from the revised primary model because it was unavailable or non-informative in the submitted analysis package.
n_S_candidates	numeric	candidate horse-level aggregation variable	Not verified / excluded	False	False	Candidate diagnostic variable excluded from the revised primary model because it was unavailable or non-informative in the submitted analysis package.
n_A_candidates	numeric	candidate horse-level aggregation variable	Not verified / excluded	False	False	Candidate diagnostic variable excluded from the revised primary model because it was unavailable or non-informative in the submitted analysis package.
draw_adjustment_sd	numeric	candidate horse-level aggregation variable	Not verified / excluded	False	False	Candidate diagnostic variable excluded from the revised primary model because it was unavailable or non-informative in the submitted analysis package.

Supplementary Table S2b. Leakage audit

Item	Status	Details
target labels	PASS	results, payout, odds, and popularity are used only to create historical race-level labels.
prediction features	PASS	revised primary model features are constructed from pre-event race-structure variables only. Candidate horse-level aggregation variables were retained in the audit inventory but were not used in the revised primary model.
post-event variables as features	PASS	finish position, payout, race result, final odds, final popularity, and same-race outcomes are excluded from model features.
label columns present in source feature table	PASS	Label columns were present only in the source table for label construction/audit purposes and were excluded before model fitting; none entered the model feature matrix. Columns: label__finish__pos; y__place; y__win.
same-day uncertain variables	PASS	same-day horse weight, uncertain going/weather, and post-entry result information are not used.
candidate-model assessment	PASS	Candidate models were assessed using the validation split only. Because the composite outcome was nearly balanced and class weighting affected calibration, the revised primary model was prespecified as unweighted standardized logistic regression. HistGradientBoostingClassifier was retained as a flexible benchmark. Test metrics are recorded for audit only and are not used for model selection.
test split use	PASS	test metrics are recorded after model selection and are not used to choose the selected race-level diagnostic model.
horse-level score integration	PASS	race-level diagnostic output is not integrated into cal__total__score, cal__rank__score, recommendation rank, or recommendation count.

Supplementary Table S3. Temporal split manifest

Split	Period	Number of races	Role
train	2015-01-01 to 2022-12-31	26,625	model fitting only
validation	2023-01-01 to 2024-12-31	6,656	model selection and hyperparameter/model-family comparison
test	2025-01-05 to 2026-05-10	4,556	final temporal evaluation only; not used for model selection

Supplementary Table S4. Candidate model configuration and validation-only assessment

Candidate models were assessed using the validation split only. The validation-only audit score was defined as

$$S_{\text{valid}} = 0.70 \times \text{PR-AUC}_{\text{valid}} + 0.20 \times \text{ROC AUC}_{\text{valid}} - 0.10 \times \text{Brier}_{\text{valid}}.$$

Higher values indicate better validation performance. Because the composite race-level upset outcome was nearly balanced and class weighting affected calibration, the revised primary model was prespecified as unweighted standardized logistic regression. HistGradientBoostingClassifier was retained as a flexible benchmark. Test metrics in this table were recorded for audit and were not used for model selection.

Model	Primary	Selection score	Valid ROC AUC	Valid PR-AUC	Valid Brier	Test ROC AUC recorded only	Test PR-AUC recorded only	Test Brier recorded only	Assessment policy
logistic__unweighted	True	0.5417	0.6396	0.6245	0.2341	0.6459	0.6308	0.2325	Prespecified revised primary model: unweighted standardized logistic regression using race-structure variables. Test metrics are recorded for audit only.
logistic__balanced	False	0.5417	0.6396	0.6246	0.2348	0.6460	0.6309	0.2326	Class-weight sensitivity model. Not selected as primary because class weighting did not materially improve discrimination and affected calibration.
hist__gradient__boosting	False	0.5411	0.6406	0.6234	0.2339	0.6500	0.6369	0.2315	Nonlinear benchmark model. Test metrics are recorded for audit only and are not used for model selection.

Supplementary Table S5. Calibration table for the temporal test split

Empty bins are retained to make the calibration grid explicit. Expected calibration error was computed as the sample-size-weighted mean absolute difference between the mean predicted probability and the observed event rate across 10 equal-width probability bins.

Split	Predicted probability bin	N	Mean prediction	Observed event rate	Absolute error
test	0.0–0.1	0			
test	0.1–0.2	29	0.1795	0.1379	0.0416
test	0.2–0.3	269	0.2627	0.1784	0.0842
test	0.3–0.4	517	0.3523	0.3443	0.0080
test	0.4–0.5	715	0.4523	0.4601	0.0078
test	0.5–0.6	1,450	0.5572	0.5248	0.0323
test	0.6–0.7	1,373	0.6388	0.6453	0.0065
test	0.7–0.8	198	0.7302	0.6919	0.0382
test	0.8–0.9	5	0.8109	0.8000	0.0109
test	0.9–1.0	0			

Supplementary Table S6. Upset-risk stratification coverage results in the temporal test set

Event-rate uncertainty was summarized using 1,000 race-level bootstrap resamples with `race_id` as the resampling unit.

Direction	Coverage	N	Events	Event rate	Baseline	Lift	95% bootstrap CI	Bootstrap unit
high_upset_top_risk	5%	228	156	0.6842	0.5151	1.33	0.6184–0.7412	race_id
low_upset_bottom_risk	5%	228	36	0.1579	0.5151	0.31	0.1096–0.2105	race_id
high_upset_top_risk	10%	456	311	0.6820	0.5151	1.32	0.6403–0.7259	race_id
low_upset_bottom_risk	10%	456	100	0.2193	0.5151	0.43	0.1754–0.2544	race_id
high_upset_top_risk	20%	911	608	0.6674	0.5151	1.30	0.6355–0.6971	race_id
low_upset_bottom_risk	20%	911	268	0.2942	0.5151	0.57	0.2634–0.3227	race_id
high_upset_top_risk	30%	1,367	894	0.6540	0.5151	1.27	0.6291–0.6767	race_id
low_upset_bottom_risk	30%	1,367	488	0.3570	0.5151	0.69	0.3285–0.3833	race_id
high_upset_top_risk	40%	1,822	1,172	0.6432	0.5151	1.25	0.6202–0.6641	race_id
low_upset_bottom_risk	40%	1,822	721	0.3957	0.5151	0.77	0.3743–0.4199	race_id
high_upset_top_risk	50%	2,278	1,401	0.6150	0.5151	1.19	0.5939–0.6352	race_id
low_upset_bottom_risk	50%	2,278	947	0.4157	0.5151	0.81	0.3964–0.4355	race_id

Supplementary Table S7. Component-level outcome performance for the revised race-level diagnostic score

This table reports temporal test-set performance when the same revised race-level diagnostic score was evaluated against each component label and the composite upset label. Values in brackets indicate 95% confidence intervals estimated using race-level bootstrap resampling.

Outcome	Metric	Estimate	95% bootstrap CI
target_upset_win	Positive rate	0.2395	–
target_upset_win	ROC AUC	0.6030	0.5863–0.6210
target_upset_win	PR-AUC	0.3030	0.2836–0.3265
target_upset_win	Brier score	0.2659	0.2620–0.2693
target_upset_win	Log loss	0.7257	0.7176–0.7330
target_upset_win	ECE-10	0.2906	0.2792–0.3025
target_upset_win	Calibration intercept	-1.2873	-1.3665 to -1.2167
target_upset_win	Calibration slope	0.7498	0.6259–0.8871
target_upset_place	Positive rate	0.4339	–
target_upset_place	ROC AUC	0.6478	0.6326–0.6645
target_upset_place	PR-AUC	0.5532	0.5311–0.5767
target_upset_place	Brier score	0.2377	0.2339–0.2411
target_upset_place	Log loss	0.6673	0.6594–0.6746
target_upset_place	ECE-10	0.0961	0.0821–0.1099
target_upset_place	Calibration intercept	-0.4313	-0.4955 to -0.3710
target_upset_place	Calibration slope	1.1093	0.9857–1.2507
target_high_payout_revised	Positive rate	0.1826	–
target_high_payout_revised	ROC AUC	0.6721	0.6538–0.6902
target_high_payout_revised	PR-AUC	0.2874	0.2644–0.3147
target_high_payout_revised	Brier score	0.2638	0.2600–0.2672
target_high_payout_revised	Log loss	0.7211	0.7129–0.7283
target_high_payout_revised	ECE-10	0.3475	0.3376–0.3579
target_high_payout_revised	Calibration intercept	-1.8206	-1.9207 to -1.7317
target_high_payout_revised	Calibration slope	1.3759	1.2159–1.5637
target_race_upset_revised	Positive rate	0.5151	–
target_race_upset_revised	ROC AUC	0.6459	0.6305–0.6628
target_race_upset_revised	PR-AUC	0.6308	0.6097–0.6526
target_race_upset_revised	Brier score	0.2325	0.2288–0.2359
target_race_upset_revised	Log loss	0.6567	0.6491–0.6639
target_race_upset_revised	ECE-10	0.0213	0.0154–0.0371
target_race_upset_revised	Calibration intercept	-0.0730	-0.1383 to -0.0122
target_race_upset_revised	Calibration slope	1.0663	0.9525–1.1938

Supplementary Table S8. Benchmark and ablation analysis for race-structure feature sets

This table reports validation and temporal test performance for simple race-structure benchmark and ablation feature sets. Test metrics were recorded for audit and were not used for model selection.

Feature set	Valid ROC AUC	Valid PR-AUC	Valid Brier	Test ROC AUC	Test PR-AUC	Test Brier	Test ECE-10
intercept_only	0.5000	0.5158	0.2501	0.5000	0.5151	0.2502	0.0204
field_size_only	0.6160	0.5908	0.2372	0.6220	0.5933	0.2357	0.0242
race_no_only	0.5346	0.5437	0.2494	0.5450	0.5510	0.2491	0.0204
course_surface_only	0.5404	0.5458	0.2490	0.5422	0.5443	0.2490	0.0201
distance_only	0.5215	0.5292	0.2497	0.5450	0.5474	0.2487	0.0186
class_only	0.5224	0.5357	0.2493	0.5354	0.5451	0.2490	0.0223
race_structure_core	0.6382	0.6243	0.2342	0.6466	0.6295	0.2325	0.0234
race_structure_full_without_year	0.6396	0.6245	0.2341	0.6459	0.6308	0.2325	0.0213
race_structure_full_with_year	0.6393	0.6243	0.2344	0.6456	0.6307	0.2329	0.0266

Supplementary Table S9. Software versions and reproduction commands

All analyses were conducted in Python 3.13.7 using pandas 3.0.2, NumPy 2.4.4, and scikit-learn 1.8.0. The primary random seed was fixed at 2026. Representative reproduction commands for the revised analysis package are shown below in monospaced font.

Step	Command
Out-of-sample gate audit	& "<python_3_13_executable>" .\scripts\08_research_eval\research2_blocking_issue_oos_gate.py
Revised race-structure analysis	& "<python_3_13_executable>" .\scripts\08_research_eval\research2_b_plan_race_structure_revision.py
Regenerate Figure 2	& "<python_3_13_executable>" .\scripts\08_research_eval\research2_b_plan_make_figure2_component.py
Compile checks	& "<python_3_13_executable>" -m py_compile .\scripts\08_research_eval\research2_blocking_issue_oos_gate.py .\scripts\08_research_eval\research2_b_plan_race_structure_revision.py .\scripts\08_research_eval\research2_b_plan_make_figure2_component.py

Supplementary Table S10. Revision source manifest with SHA-256 hashes

This table documents the revised analysis artifacts used to build the manuscript and supplementary tables without exposing local directory structure. Licensed raw data and row-level processed datasets are not redistributed.

Artifact	Category	Exists	Size bytes	SHA-256
research2__blocking__issue__oos__gate.py	source script	True	40727	6206d5889174833569a959ebb7803a96650779e8217edac7e3b3e3f1da7c5fb8
research2__b__plan__race__structure__revision.py	source script	True	58070	f02d73d9361af5d39bc544400fcbc5aec40adc5a27843aa1b042e25d2d9bc758
research2__b__plan__make__figure2__component.py	source script	True	4821	325822b5bf302b272ae55958de789b0dc628d6da3bf5eb9690af8600c081d9e9
REVISE__REVISION__AUDIT__LOG.md	revision documentation	True	831	e97824392b026be75eb64e936c1ba74b997722aebd3b8bbcbd59be046276005a
REVISE__REVISION__ACTION__PLAN.md	revision documentation	True	1491	145cbf27266bb3bb08132234b0a1cd825984c956add5652d44825f0ad33aae0f
REVISE__MANUSCRIPT__REWRITE__MAP.md	revision documentation	True	1571	86ddf9f50a927c451813f0b1d0cab9e87cd743ac449581a24a817047e0b7d561
REVISE__MAIN__DISPLAY__ITEM__PLAN.md	revision documentation	True	702	f9a548561d2a87b26fbd9b3db29da7b3c83fc81d672fb69df3d77d22cd32161d
REVISE__SUPPLEMENTARY__STRUCTURE.md	revision documentation	True	768	e8545296978561b46ffe787c91fba3a3cb67d342115feb19fd209c65b2b4f9f8
REVISE__primary__model__performance.csv	analysis csv	True	2901	1b745d24d317973f7069d99ee1fc7120613f5a094ec8b4c7640bb7093076a357
REVISE__primary__model__bootstrap__ci.csv	analysis csv	True	3768	ef545abaf51ffd5d03515784eb58d4a12a86e09e934a2780c5dee830077572d2
REVISE__component__outcome__performance.csv	analysis csv	True	1197	ad0aa0ff7866c5945d7fb43ac6fe8b86be22597a5b3970b5b0a32b2fc3486ec7
REVISE__benchmark__feature__set__performance.csv	analysis csv	True	7750	9071b4438ef7e1607428e1f336e28151e877992eacc3d53c6429474835466038
REVISE__primary__model__coefficients.csv	analysis csv	True	7623	3dfb0ba25aff652412cd9778f291533e261c09db6d3ee27c7f09c24ffb665fd1
REVISE__component__metric__bootstrap__ci.csv	analysis csv	True	4209	12469399b81b02831f7bd32bcdb144565a52afa08fe98f0524954c5e8cdb9359
REVISE__benchmark__metric__bootstrap__ci.csv	analysis csv	True	10136	171aef5ea189a2925997c616e245f0856bcabaff57938d64c32d6edd8b3cb071
REVISE__benchmark__feature__set__differences__vs__full.csv	analysis csv	True	6332	78cb021bebc9ab4f930deee1f2b6f88bfd44dab27429ab5046769067452bb353
REVISE__calibration__summary__extended.csv	analysis csv	True	249	b282d9100474776b5769225235691807118517480e2d7e8151b99df08df269d3
REVISE__class__weight__sensitivity.csv	analysis csv	True	1987	83d03be0b37546962da7e963784ea0da545e21ffbf0c6c24e4f4dd6aaf525c30
REVISE__selective__coverage__bootstrap__ci.csv	analysis csv	True	1847	1c36e40497b24e54a1fa4a3d5eaf3d0f76891794b0a5c768cffc79789da347d5
REVISE__figure__calibration__primary.png	figure artifact	True	61899	98fcc6495a9ec58776094236041459cf05471a6f2b5316d97c2281697067b4b0
REVISE__figure__upset__risk__stratification.png	figure artifact	True	53949	36a994aa99e2fd5b9ee742e6e8177b3ce61ffe17eabe6648070c999558368f3c
REVISE__figure2__multipanel__calibration__stratification.png	figure artifact	True	123237	ea9fd21a6805ba0b1330599a769b4dc6f9b2cbd0a2970e9a8eae14a9a7f7a74d
